# Camouflaged object detection via context and texture-aware hierarchical interaction

**DOI:** 10.1038/s41598-025-32409-9

**Published:** 2026-03-18

**Authors:** Zhi Wang, Yangyang Deng, Chenxing Shen, Miaohui Zhang, Xiaoxia Lu

**Affiliations:** 1 Department of Radiology, Medical Imaging Research Institute, Huaihe Hospital of Henan University, Kaifeng, 475000 China; 2 School of Artificial Intelligence, Henan University, Zhengzhou, 450046 China; 3 School of Nursing and Health, Henan University, Kaifeng, 475000 China

**Keywords:** Camouflaged object detection, Texture encoder, Hierarchical structure, Computational biology and bioinformatics, Engineering, Mathematics and computing

## Abstract

In the field of camouflaged object detection (COD), effectively distinguishing the intrinsic similarity between objects and their backgrounds is a critical factor for improving detection performance. Existing approaches typically leverage boundary constraints to provide additional auxiliary information during the training phase. To capture more discriminative detailed cues, we introduce texture labels as supervisory signals and propose a context- and texture-aware hierarchical interaction network (CTHINet) for COD. In the coding phase, the network is divided into two separate branches, a context and a texture encoder. Specifically, a context encoder is employed to generate contextual information. Subsequently, the features at different scales are refined by implementing a Multi-head Feature Aggregation Module (MFAM). The diversity of features is subsequently enhanced by leveraging the interactions among their distinct feature receptive fields, facilitating the matching of candidate areas for camouflaged objects with varying sizes and shapes. Following this, the enhanced features are combined with texture features generated by the texture encoder, fully exploiting imperceptible cues within candidate objects through utilizing the Hierarchical mixed-scale Interaction Modules (HMIM). This module continuously integrates texture cues with contextual information within a single feature scale, aiming for more accurate detection. Extensive experiments conducted on three challenging benchmark datasets, e.g., CAMO, COD10K, and NC4K, illustrate that our model has superior performance compared to state-of-the-art methods. Furthermore, the evaluation results on the polyp segmentation dataset underscore the promising potential of CTHINet for downstream applications.

## Introduction

Camouflage is a prevalent phenomenon in both the natural and human worlds. It serves as a survival strategy for creatures in nature, allowing them to hide from predators. In the human world, camouflage is often employed for artistic endeavors and military activities. Camouflaged object detection (COD) is designed to identify objects that resemble their environment in terms of color, texture, and shape. The high intrinsic similarities between them pose a greater challenge to camouflaged object detection than traditional object detection. Nevertheless, camouflaged object detection remains a topic of significant research interest due to its diverse applications in various fields, including medical image processing (polyp segmentation^1^, lung infection segmentation^2^), industry (surface defect detection^3^), and marine fisheries (underwater object detection^4^).

There is a long history of research on camouflaged object detection. Traditional methods have attempted to differentiate camouflaged objects from their surroundings through the use of handcrafted low-level features (e.g., motion^5^, texture^6^, edge^7^, and 3D convexity^8^). Owing to the limited ability of handcrafted features to distinguish between background and foreground, and the inability to effectively detect intra-class differences, these methods often exhibit subpar performance in complex scenes.

Recently, with the release of several independent benchmarks and the rapid development of deep learning, there has been significant progress in camouflaged object detection, demonstrating substantial potential. In 2019, Le et al.^9^ suggested an anabranch network where the second branch is dedicated to predicting the presence of a camouflaged object in an image. This information is subsequently integrated into a camouflaged object segmentation task. The first camouflaged object detection dataset, CAMO, was also created. However, due to the limited sample data, it could not take full advantage of the deep learning model. In 2020, the first large-scale public dataset, COD10K was released by Fan et al.^10^. Drawing inspiration from the hunting process, they proposed a two-stage network to accurately detect camouflaged objects. The network involves acquiring candidate features through the search module and subsequently processing them through the identification module, achieving commendable results and significantly propelling the development of COD. In 2021, Lv et al.^11^ presented the largest test dataset, NC4K, comprising 4121 images, for comprehensive model evaluation. They introduced a framework designed to simultaneously address the triple task of localizing, segmenting, and ranking camouflaged objects.

**Fig. 1:**
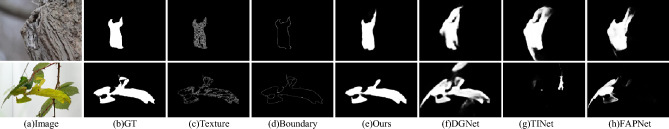
Illustrations of representative camouflage situations observed in the natural world, along with the corresponding detection results obtained through various methods. In the natural world, camouflage is primarily achieved through background matching, where camouflaged objects often exhibit indistinguishable textures and blurred boundaries. Ground truth (GT) (**b**) is a binary map representing the camouflaged objects. Texture (**c**) corresponds to the texture label of the source image. Boundary (**d**) depicts the outline of the camouflaged objects. Additionally, (**e**–**h**), represent the COD results of these two images using different methods. (The images are sourced from publicly available COD datasets: COD10K (https://github.com/DengPingFan/SINet/), NC4K (https://github.com/JingZhang617/COD-Rank-Localize-and-Segment), and CAMO (https://sites.google.com/view/ltnghia/research/camo). All datasets are available for non-commercial use, and only require citation of the corresponding papers).

Unlike salient object detection (SOD), camouflaged object detection (COD) requires a more extensive cognitive process to overcome adversarial deception^12^. With the advancement of related research, the adoption of additional auxiliary cues to facilitate recognition has gradually become prevalent, such as boundary-based^13,14^ and uncertainty-guided^15^ approaches, aiming to enhance the discriminative underlying representations for COD. In complex scenes, boundary-supervised or uncertainty learning often tends to overemphasize the sparse edges of camouflaged objects, leading to the introduction of noisy features. This challenge arises from the difficulty in accurately delineating the edges of camouflaged objects. In contrast, despite the object’s best efforts to camouflage, subtle clues are invariably left behind. These clues are texture information, which has already found extensive application in other fields^16,17^. To capture these clues, texture information has been more frequently integrated into the field of camouflaged object detection^18,19^. As shown in Fig. 1, compared with boundary supervision, texture supervision more effectively mitigates sparsity issues and suppresses noisy inputs, thereby guiding the model to focus on intrinsic fine-grained image details^20,21^. Moreover, studies have demonstrated the significance of contextual information in enhancing the accuracy of small object detection^22^ and object detection under occlusion^23^.

Building upon the considerations mentioned above, we propose a novel network. To streamline the learning process, we decompose the network into two interconnected branches, namely, context and texture encoders. The former is dedicated to acquiring contextual semantics, while the latter focuses on learning texture information. This approach avoids interfering between texture branches and context branches. Specifically, for the context encoder, we employ the improved Pyramid Vision Transformer (PVTv2)^24^ for extracting multi-scale global contextual information. Meanwhile, the texture encoder leverages texture labels as supervisory signals via convolutional neural networks (CNNs) to optimize the feature extraction process. In this paper, we introduce a Multi-head Feature Aggregation Module (MFAM). In contrast to existing models, this module divides the features into four heads along the channel, with each head dedicated to a convolution using a different convolutional kernel size. This design allows the module to capture multi-scale features and extend the receptive field while facilitating information fusion across other heads. This capability is crucial for matching candidate areas of camouflaged objects with different sizes and shapes, thereby contributing to model performance improvement. In addition, we introduce the Hierarchical mixed-scale Interaction Modules (HMIM) based on the Group Aggregation Bridge (GAB). This module is specifically designed to integrate texture information with contextual information effectively. The GAB module reorganizes and enhances the output features from the context encoder and texture encoder along the channel dimension to effectively achieve organic information integration. To enhance the representation of individual features, we propose a hierarchical model using GAB. To select critical features more accurately, we incorporate the channel attention mechanism. This mechanism contributes to creating a more nuanced HMIM by re-weighting the features. In summary, our contributions are as follows:

1. We propose a Multi-head Feature Aggregation Module (MFAM). Specifically, this module adopts a multi-head architecture that leverages information interaction across distinct feature receptive fields, enabling adaptive matching of camouflaged candidate regions with varying sizes and shapes.
2. Hierarchical mixed-scale Interaction Modules (HMIM) is proposed to fully exploit the more discriminative texture features derived from texture encoders. In this module, the GAB within the hierarchical structure reorganizes and enhances two different sets of information in the channel dimension. Subsequently, the HMIM maintains the most valuable information through feature re-weighting, resulting in accurate COD.
3. Experimental results show that our network performs better than state-of-the-art (SOTA) methods across three COD benchmark datasets. Additionally, we use the polyp segmentation dataset as an example to showcase the applicability of the proposed method to downstream applications.

The remainder of the paper will be organized as follows. The research closely related to our study is reviewed in Sect. “Related work”. Section “Proposed framework” describes our CTHINet network in detail. Section “Experiments” presents experimental results to validate the effectiveness of our method and demonstrates its application to polyp segmentation. Section “Conclusion” summarizes the paper.

## Related work

### Camouflaged object detection

Traditional methods in COD primarily utilize handcrafted low-level features, including optical flow^25^, covariance matrix^26^, and 3D concavity^8^, to differentiate camouflaged targets from the background. The performance of these methods degrades dramatically in complex backgrounds. With the emergence of relevant datasets, Various COD techniques based on deep learning have surfaced. These methods can be broadly categorized into several types. (a) Bionic method: Fan et al.^10^ introduced an effective network for COD called SINet. Drawing inspiration from animal hunting strategies, the model is structured into a search module and an identification module. Pang et al.^27^ introduced ZoomNet, a mixed-scale triple network that simulates human behavior when viewing blurred images. Its network inputs encompass images of different sizes simultaneously. Zhang et al.^28^ delved into the intrinsic mechanisms of predatory behavior and introduced PreyNet. The model simulates the entire process of predation through initial detection and predator learning. (b) Attention mechanism: Mei et al.^29^ introduced PFNet, a model based on a distraction mining strategy. This approach involves initially locating the camouflaged object and subsequently filtering out redundant interference through distraction mining. Sun et al.^30^ designed a context-aware cross-layer fusion network that aggregates multi-layer features via attention-guided cross-layer feature fusion. Zhuge et al.^31^ proposed a cube-like COD architecture that is accompanied by attentional fusion and x-shaped connections to integrate multilayer features fully. (c) Assisted Joint Learning: Le et al.^9^ proposed an end-to-end COD network by introducing an additional branch to gather classification information and integrate it into the segmentation stream. Lv et al.^11^ introduced a multi-task COD network capable of simultaneously localizing, segmenting, and ranking camouflaged objects. In addition, there is the JCSOD^15^ model, which uses salient object learning to camouflage objects. (d) Transformer or graph structure: Zhai et al.^13^ utilized graph structure to decouple features into two interrelated tasks. This model enhances features with mutual graph learning to detect camouflaged objects with more complete spatial structure details. Zhang et al.^32^ proposed a transformer-induced progressive refinement network, TPRNet. This network aggregates rich semantic information through transformers and interacts with low-level features to obtain rich fine-grained clues. Additionally, Yang et al.^33^ introduced UGTR, integrating a probabilistic representation model with transformers to implement COD with uncertainty learning. Zhong et al.^34^ employed a transformer to extract valuable information related to COD in the frequency domain.

Recently, Ji et al.^19^ introduced a deep gradient network camouflage object detection method. This approach utilizes gradient-induction transition to establish connections between contextual and texture features, consequently elevating COD performance to a new level. Furthermore, it has been widely demonstrated that employing effective feature aggregation methods^35,36^ can significantly enhance performance across numerous visual tasks. In contrast to the above COD methods, our proposed CTHINet mitigates the ambiguity in feature extraction through a dedicated branch design. Meanwhile, by adopting multi-head convolutions, it facilitates information interaction between different convolutional kernels within the same layer. Furthermore, through a hierarchical structural design, the network achieves effective fusion of high-level semantic features.

### Vision transformer

The Transformer was initially introduced by Vaswani et al.^37^ as a tool for natural language processing, gained further attention in computer vision research. Dosovitskiy et al.^38^ proposed the first transformer model for the computer vision community, known as ViT. This model explores remote spatial correlations by using a sequence of image patches directly as input for classification tasks. Subsequently, various ViT-based variants have emerged, demonstrating notable advancements in diverse vision applications, including image classification^39^, object detection^40^, and semantic segmentation^41^. Attributed to the self-attention mechanism, the Transformer excels in capturing remote dependencies, outperforming CNN-based models. However, utilizing a Transformer comes with significant computational and memory costs. To address this challenge, Wang et al.^24^ introduced the feature pyramid structure into ViT and proposed the Pyramid Vision Transformer method, which effectively mitigates the computational burden of the network.

### COD-related vision tasks

COD has many potential applications, typical examples including polyp segmentation^1^, COVID-19 lung infection segmentation^2^, and defect detection^3^. Studies on polyp segmentation play a crucial role in aiding physicians in identifying polyps from colonoscopy images. This information is invaluable for accurate diagnosis and surgical planning. Traditional methods for polyp segmentation primarily depend on texture and geometric features^42,43^. However, these methods often yield segmentation results of lower quality. As deep learning plays an increasingly prominent role in medical image analysis, there has been a rapid advancement in polyp segmentation in recent years. Notably, the model proposed by Akbari et al.^44^, which utilizes fully convolutional networks demonstrating superior performance compared to traditional methods. Furthermore, encoder-decoder-based architectures like U-Net^45^ and UNet + + ^46^ have emerged as dominant players in this field, showcasing exceptional performance. In contrast to previous approaches, Fan et al.^1^ employ a parallel partial decoder to generate global feature maps from high-level features. Subsequently, they utilize a reverse attention module to establish relationships between regions and boundary cues. In this study, we showcase the effectiveness of CTHINet by applying it to the task of polyp segmentation.

## Proposed framework

### Overall architecture

**Fig. 2:**
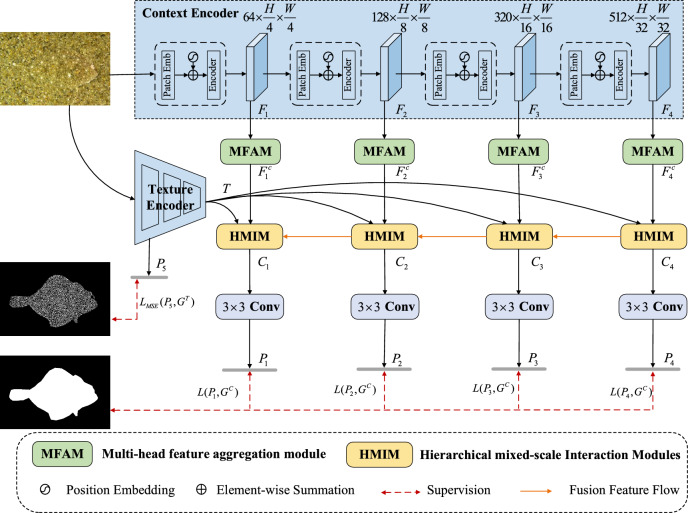
The overall framework of the proposed CTHINet. The framework is divided into two branches: the texture encoder and the context encoder. We employ PVTv2^24^ as the context encoder and construct the texture encoder using several consecutive convolutional blocks. The output features of the context encoder are delivered to the MFAM to refine them individually, aiming to capture rich multi-scale contextual information in each surrounding layer. Subsequently, the texture and context features are fed through the HMIM module to aggregate features. The network follows a coarse-to-fine structure to enhance the camouflaged features progressively. (The images are sourced from the publicly available COD dataset: CAMO (https://sites.google.com/view/ltnghia/research/camo).

Figure 2 illustrates the overall framework of our camouflage object detection model, CTHINet. First, given an RGB image $I \in {\mathbb{R}}^{3 \times H \times W}$, we utilize the PVTv2 network backbone to extract 4-layer features $F_{k} ,k \in \{ 1,2,3,4\}$. Additionally, a texture encoder module is designed to extract relevant texture cues from the original image. It consists of four stacked ConvBR(denoting the standard convolutional layer followed by a normalization layer and an activation layer) layers. Given the input image $I \in {\mathbb{R}}^{3 \times H \times W}$, it undergoes sequential processing through the first and second ConvBR layers, featuring convolutional kernel sizes of $7 \times 7$ and $3 \times 3$, respectively, and output channels set to 64. Subsequently, it passes through the third ConvBR layer with a convolutional kernel size of $3 \times 3$ and an output channel of 32. The resulting feature map from this layer is denoted as $T \in {\mathbb{R}}^{{32 \times H_{g} \times W_{g} }} ,H_{g} = H/8,W_{g} = W/8$, which will be involved in the feature fusion process of HMIM. Subsequently, it undergoes a ConvBR layer with a $1 \times 1$ convolutional kernel and a channel of 1, and the resulting output will then be combined with the texture label to compute a loss, which optimizes the texture encoder. The PVTv2 backbone progressively extracts features from low to high levels. Therefore, we adopt a coarse-to-fine modeling structure, where features $F_{k}$ are input into the corresponding MFAM to enhance the backbone features, resulting in new features $F_{k}^{c} \in {\mathbb{R}}^{{64 \times (H/2^{k + 1} ) \times (W/2^{k + 1} )}}$. Subsequently, the texture feature $T$, extracted by the texture encoder, and the enhanced context feature $F_{k}^{c}$ are fed into the HMIM to complete the modulation between different features, resulting in the feature $C_{k}$. After that, a $3 \times 3$ convolutional layer processes this feature to generate the segmentation result map. The ultimate detection results are acquired through a stepwise reconstruction involving the four HMIM modules. The network is trained using a multilevel supervised strategy.

### Multi-head feature aggregation module

**Fig. 3:**
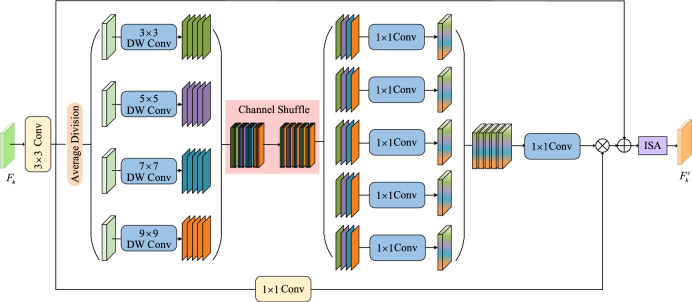
The architecture of our multi-head feature aggregation module.

Camouflaged objects often exhibit significant variations in appearance, including scale changes, occlusion, and blurred boundaries. This information serves as a crucial cue, aiding in differentiating camouflaged objects from their surrounding environments. Introducing convolutional kernels of various sizes enables the network to learn to capture features of objects at different scales. However, including large kernel convolutions inevitably leads to increased computational requirements and a higher number of parameters. Therefore, we propose the MFAM, which adopts the depth-wise separable convolution. Specifically, by integrating a multi-head architecture that leverages information interaction across distinct feature receptive fields, the module achieves adaptive matching of camouflaged candidate regions with varying sizes and shapes. This design effectively enhances the contextual information captured by the PVTv2^24^ backbone at each scale. As illustrated in Fig. 3, to standardize the number of channels in the input feature map $F_{k}$, we initially employ a $3 \times 3$ convolution for adjusting the channel number. Subsequently, it is divided into n groups along the channel dimensions, with each group by a distinct depth-wise separable convolution with a unique kernel size. To simplify the design process, we initialize with $3 \times 3$ kernel size and increment each group sequentially by 2. Once the number of groups is established, the convolutional kernel size for each group is also determined. We can adjust the range of receptive field and multi-granularity information by adjusting the amount of grouping. This portion of the feature $X$ can be represented as:

1 $$X = Concat(DW_{{m_{1} \times m_{1} }} (F_{k}^{1} ),...,DW_{{m_{n} \times m_{n} }} (F_{k}^{n} ))$$

where $F^{\prime}_{k} = [F_{k}^{1} ,F_{k}^{2} ,...,F_{k}^{n} ]$ implies that there are n groups of input features in the channel dimension. In this paper, n is set to 4. $F_{k}^{\prime }$ denotes the input feature channel size is reduced to 64 with a $3 \times 3$ convolutional operation, and $m_{i} \in \{ 3,5,...,M\}$ denotes the kernel size monotonically increasing by 2 per head.

However, our use of different-sized convolutions for each feature group did not account for information interaction between groups. To address this limitation, we introduce a new lightweight aggregation module, facilitating more comprehensive interaction among information from different groups. First, we subjected the resulting feature $X$ with different granularities to channel shuffle operations and regrouping. Specifically, we create a new group by selecting one channel from each existing group, ensuring that features in each new group represent all groups. Subsequently, we employ the inverse bottleneck structure to conduct feature fusion within each group using a $1 \times 1$ convolution. The outputs of each group are then passed to the final $1 \times 1$ convolution for feature fusion after concatenation along the channel axis. This approach enhances the diversity of multi-scale features. At this point, we have obtained an output feature mapping $M$. Following that, we apply $1 \times 1$ convolution to the features represented by $F_{k}^{\prime }$ to calculate the modulation values $V$. We multiply the feature mapping $M$ and the modulation values $V$, utilizing point-by-point convolution to aggregate information across all feature groups.

To further improve the integrated features and enhance their discriminative power, we employ the ISA^47^ scheme instead of the convolution operation. Before this, we introduce residual learning. ISA decomposes the task into the product of two attention, long-range and short-range. ISA achieves better results than the original Non-local module with less computational and memory complexity. Specifically, the ISA used in MFAM has a descent factor of (8,8).

### Hierarchical mixed-scale interaction modules

The background and the object that is concealed have subtle texture differences. To this end, we propose an adaptive fusion method via a hierarchical structural design to explore the potential correlations between contextual and textural features, as their interaction is crucial. Common interactions from previous approaches, such as multiplication-summation^48^ and soft grouping^19^, have been widely adopted. For multiplication-summation, texture features are employed to modulate contextual features, facilitating feature enhancement. Additionally, soft grouping provides parallel nonlinear projections at multiple fine-grained subspaces. Unlike previous approaches, we aim to construct an effective interaction module that enhances the nuance between the object and the background.

**Fig. 4:**
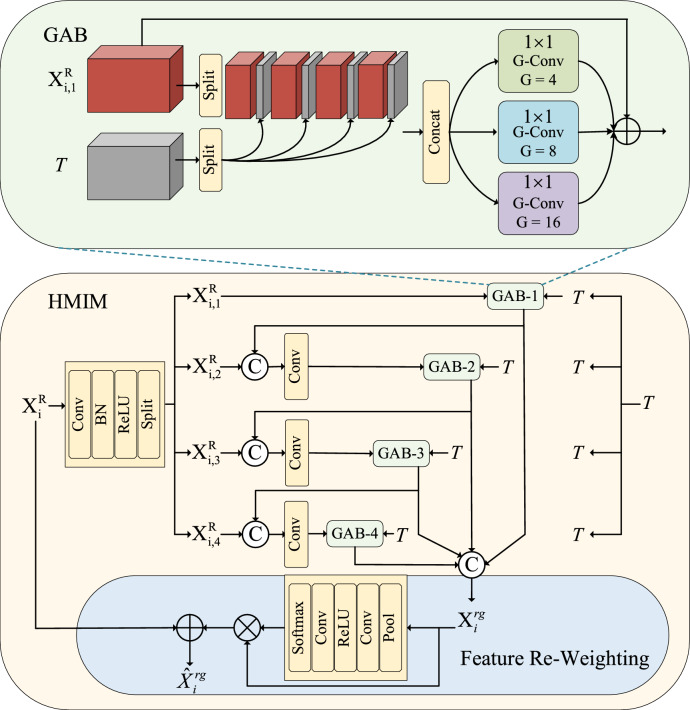
Illustration of the HMIM. It realizes an effective fusion between texture features and contextual features. HMIM consists of three main components: hierarchical branching, group aggregation bridge, and feature re-weighting operations.

As shown in Fig. 2, the HMIM is divided into two different inputs. The first type of input contains only the multi-scale fusion features from the MFAM and texture features. The second type of input includes the outputs from the higher-level HMIM, in addition to the above two inputs. For the second type of input, we first concatenate the input from MFAM and the higher-level HMIM, along the channel dimensions. After passing through a convolutional compression channel, we sum it up with the original two feature elements. This process ensures that its multi-scale fusion features are uniformly represented as $X_{{\mathrm{i}}}^{R} ,i \in \{ 1,2,3,4\}$. As shown in Fig. 4, within the input stage of HMIM, we adopt $1 \times 1$ convolution to expand the channel number of the feature map $X_{i}^{R}$. Subsequently, we divide the feature into G groups $\{ X_{{_{i,j} }}^{R} \}_{j = 1}^{G}$ along the channel dimensions, with one branch in each group. Starting from the initial branch, the output of the preceding GAB will participate in the processing of features in the subsequent GAB, called progressive fusion. Hierarchical structures prove particularly effective for HMIM, enabling the generation of features enriched with contextual information. Specifically, the input is segregated into a context feature and a texture feature. We further partition these two features into fixed groups along the channel dimension, denoted as follows:

2 $$\begin{gathered} T \in {\mathbb{R}}^{{C_{g} \times H_{g} \times W_{g} }} \to \{ T^{m} \}_{{{\mathrm{m}} = 1}}^{M} \in {\mathbb{R}}^{{K_{g} \times H_{g} \times W_{g} }} \hfill \\ X_{i,j}^{R} \in {\mathbb{R}}^{{C_{i} \times H_{i} \times W_{i} }} \to \{ X_{i,j}^{R,m} \}_{{{\mathrm{m}} = 1}}^{M} \in {\mathbb{R}}^{{K_{i} \times H_{i} \times W_{i} }} \hfill \\ \end{gathered}$$

where $\to$ denotes the grouping operation. The channel number of each feature group is denoted by $K_{i} = C_{i} /M$ and $K_{g} = C_{g} /M$, while the number of groups is represented by $M = 8$.

The grouped features are alternately spliced between the two groups in the channel dimension to generate the regrouped feature $Q_{j} \in {\mathbb{R}}^{{(C_{i} + C_{g} ) \times H_{i} \times W_{i} }}$, the total channel number is the sum of the channels in the context feature and the texture feature. To facilitate interaction between context and texture features while preserving their correlation and independence, we introduce parallel nonlinear projections in multiple fine-grained subspaces. This allows the network to take full advantage of the multi-source representation to maximize performance. We employ group convolution in parallel, using a different number of groups for each branch and fusing them. Denoted as:

3 $$A_{j} = \phi_{1} (Q_{j} ) + \phi_{2} (Q_{j} ) + \phi_{3} (Q_{j} )$$

where $\phi$ represents the group convolution, we further introduce residual learning. At this stage, we define the complete function representation as:

4 $$X_{j} = \tau_{j} (X_{i,j}^{R} ,T) = X_{i,j}^{R} \oplus A_{j}$$

As shown in Fig. 4, the previous feature will contribute to the next modulation feedback processing. This progressive approach enhances the effective fusion of local and global features, thereby increasing interaction between different branches. So, at the j-th branch, its features should be rewritten as follows:

5 $$X_{{\mathrm{i,j}}}^{R} = \left\{ \begin{gathered} X_{{\mathrm{i,1}}}^{R} ,{\text{ j}} = 1 \hfill \\ conv(cat(X_{{\mathrm{i,j}}}^{R} ,X_{j - 1} )),{\text{ j}} = 2,3,4 \hfill \\ \end{gathered} \right.$$

After completing the modulation feedback processing for all branches, we integrate the features from the four GABs to obtain contextual features as follows:

6 $$X_{i}^{rg} = concat(X_{1} ,X_{2} ,X_{3} ,X_{4} )$$

To further efficiently filter features and retain the most valuable information, we introduce an adaptive feature reweighting operation to differentially focus on four sets of enhanced features. We obtain the more valuable feature $\hat{X}_{i}^{rg}$ by utilizing a small convolutional network to modulate $X_{i}^{rg}$ in a channel-wise manner. The channel-wise feature reweighting operation can be formulated as:

7 $$\hat{X}_{i}^{rg} = CA(X_{i}^{rg} ) \odot X_{i}^{rg}$$

This significantly enhances the flexibility of our adaptive approach. Additionally, we introduce residual learning to add fused features to the input context features, resulting in the final integrated features. The proposed HMIM effectively integrates context and texture information to provide cues for COD.

### Loss function

Our model incorporates two types of supervision: object-level supervision and texture supervision. For object-level supervision, we utilize the weighted binary cross-entropy loss($L_{BCE}^{\omega }$) and weighted IOU loss($L_{IOU}^{\omega }$). These focus more on hard pixels rather than assigning equal weight to all pixels. Eventually, we combine the two loss functions as:

8 $$L = L_{BCE}^{\omega } + L_{IOU}^{\omega }$$

We use the standard mean squared error loss function for texture supervision. The proposed model has five outputs for supervision, with each HMIM producing a prediction map (denoted as $P_{1} ,P_{2} ,P_{3} {\text{ and }}P_{4}$) for object-level supervision, while the texture branch outputs $P_{5}$ for texture supervision. The final total loss function is:

9 $$L_{total} = \sum\limits_{i = 1}^{4} {L(P_{i} ,G^{C} ) + \lambda L_{MSE} } (P_{5} ,G^{T} )$$

where $G^{C}$ denotes the object-level ground truth and $G^{T}$ denotes the texture ground truth, and we set $\lambda$ to 4 in the experiment.

## Experiments

In this section, we first present our experiments in detail, encompassing model training details, as well as the datasets and evaluation metrics employed in the experiments. Then, the quantitative and qualitative comparison is made between the proposed and existing COD methods. Further, an ablation study is conducted to validate the effectiveness of the key components. Finally, we validated the model’s ability to segment polyp images.

### Experimental settings

#### Implementation details

The proposed model is implemented by the PyTorch framework and accelerated computations with RTX 3090 GPU. The context encoder is loaded with pre-trained model weights, while the remaining modules are initialized randomly. During the training process, we first resized the image to $384 \times 384$ pixels and applied four data augmentation techniques: horizontal flipping, random cropping, color enhancement, and random rotation. The model was trained using the Adam optimizer^49^ with the initial learning rate set to $8{\text{e - 5}}$. The learning rate was adjusted using the cosine annealing strategy with a maximum of 20 adjustment iterations. The total number of training epochs was 100, with a batch size of 16. During the testing phase, the input image is resized to $384 \times 384$ pixels for network processing. The final output is then taken as the prediction map, and the result is resized back to its initial size without employing heuristic post-processing techniques.

#### Datasets

We performed experiments on three publicly available benchmark datasets in this study. CAMO^9^ was divided into a training set consisting of 1000 images and a test set comprising 250 images. COD10K is the largest COD dataset currently available, featuring various camouflage scenarios. The COD10K dataset^10^ consists of 5 super-classes and 69 sub-classes, with 3040 training and 2026 test images. NC4K^11^ is the largest test set for evaluating camouflage object detection models, including 4121 camouflage images from the Internet. Maintaining the same settings as in previous studies^10,19,50^, we utilized the same training and test sets.

#### Evaluation metrics

Structure measure($S_{\alpha }$)^51^, weighted F-measure($F_{\beta }^{\omega }$)^52^, mean E-measure($E_{\phi }$)^53^, and mean absolute error($M$)^54^ are four widely used evaluation metrics in camouflage object detection. These metrics evaluate the performance from various perspectives. We utilize these metrics to quantitatively assess the performance of our method against other state-of-the-art methods. The evaluation tool provided by Fan et al.^19^. Additionally, we introduce PR curves to illustrate the model’s performance, which are generated by varying thresholds within the range [0,255].

### Performance comparison

Camouflage object detection algorithms have made rapid progress with the support of datasets, eliminating the need to introduce salient object detection algorithms for expanding the comparison methods. We compare the proposed method with 22 state-of-the-art COD methods, including SINet^10^, C2FNet^30^, TINet^48^, JCSOD^15^, LSR^11^, R-MGL^13^, PFNet^29^, C2FNet-V2^55^, ERRNet^56^, TPRNet^32^, FAPNet^57^, BGNet^58^, PreyNet^28^, ZoomNet^27^, SINetV2^50^, DGNet^19^, FSPNet^59^, Camoformer^60^, MSCNet^61^, CINet^62^, SDRNet^63^, MIGNet^64^. For a fair comparison, the result maps of the FSPNet method are obtained from^59^, while the results of the remaining 20 methods are sourced from^65^. Additionally, all prediction maps were evaluated under the same protocol using identical code.

#### Quantitative analysis

**Fig. 5:**
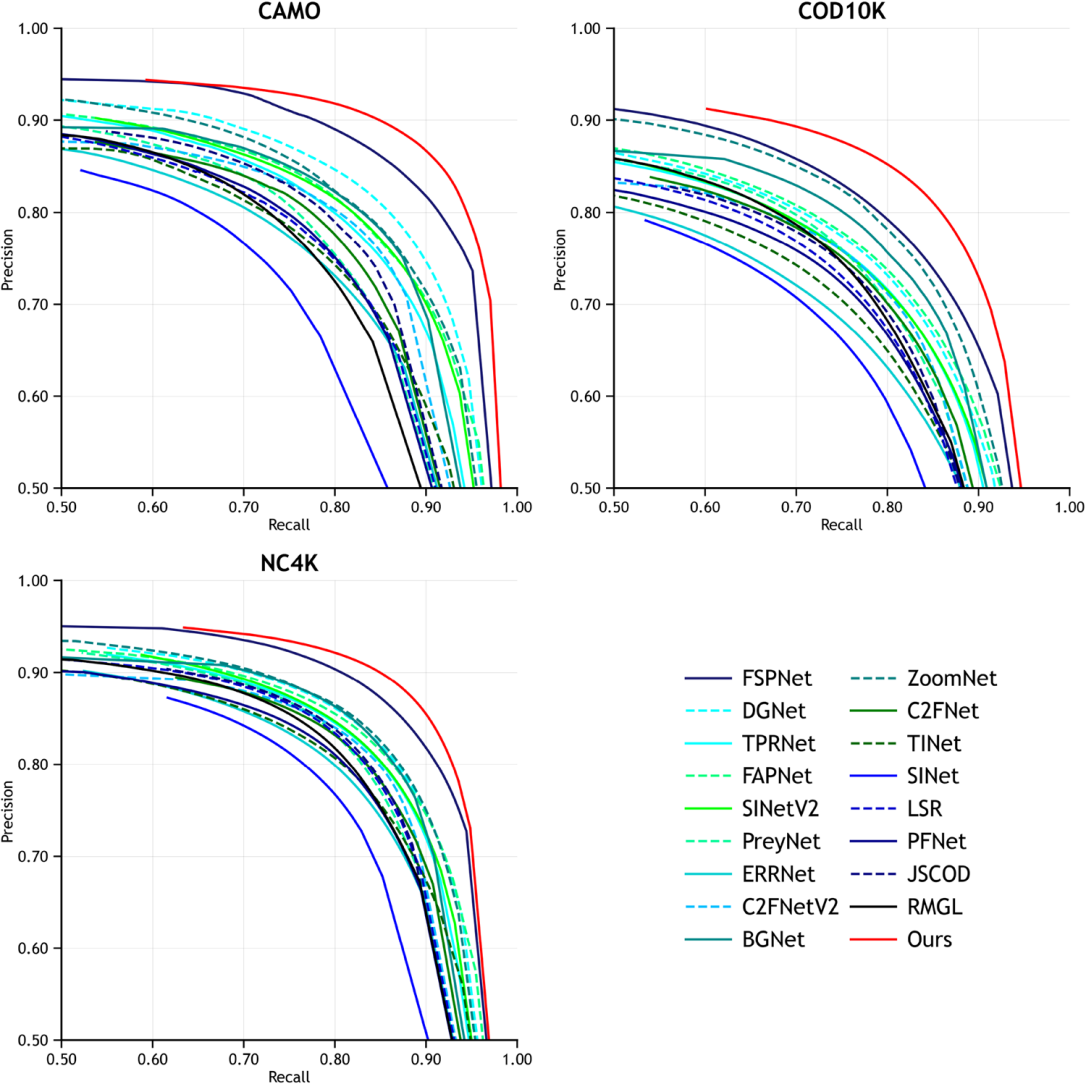
PR curves of our model and other models on the three datasets.

Table 1 summarizes the quantitative results of various COD methods using the four commonly used metrics on the three benchmark datasets. It is evident from the table that our model outperforms other methods to a significant extent, achieving impressive performance across all three datasets. Specifically, on the CAMO dataset, our model achieves superior results compared to the suboptimal FSPNet^59^ model. We observe notable improvements, with $S_{\alpha }$ and $F_{\beta }^{\omega }$ increasing by 3.0% and 5.4%, respectively, while $M$ is reduced by 12%. On the COD10K dataset, we achieved improvements of 2.1% and 6.8% for $S_{\alpha }$ and $F_{\beta }^{\omega }$, respectively, and reduced $M$ by 11.5% compared to the suboptimal FSPNet model. Meanwhile, on the largest dataset NC4K, our model achieves optimal results, indicating that the method has strong generalization capabilities. The validity of our model is further supported by the PR curve results for the three datasets shown in Fig. 5. The curves generated by CTHINet on all three datasets consistently outperform those produced by other methods, further underscoring the superiority of CTHINet over other state-of-the-art models.

**Table 1:** Results of the quantitative evaluation of different methods on three benchmark datasets using the four metrics $S_{\alpha }$, $E_{\phi }$, $F_{\beta }^{\omega }$, and $M$. “$\uparrow / \downarrow$” indicates that bigger or smaller is better.

Baseline models	CAMO				COD10K				NC4K			
	$S_{\alpha } \uparrow$	$E_{\phi } \uparrow$	$F_{\beta }^{\omega } \uparrow$	$M \downarrow$	$S_{\alpha } \uparrow$	$E_{\phi } \uparrow$	$F_{\beta }^{\omega } \uparrow$	$M \downarrow$	$S_{\alpha } \uparrow$	$E_{\phi } \uparrow$	$F_{\beta }^{\omega } \uparrow$	$M \downarrow$
SINet_20_^50^	0.745	0.804	0.644	0.092	0.776	0.864	0.631	0.043	0.808	0.871	0.723	0.058
C2FNet_21_^30^	0.796	0.854	0.719	0.080	0.813	0.890	0.686	0.036	0.838	0.897	0.762	0.049
TINet_21_^48^	0.781	0.836	0.678	0.087	0.793	0.861	0.635	0.042	0.829	0.879	0.734	0.055
JCSOD_21_^15^	0.800	0.859	0.728	0.073	0.809	0.884	0.684	0.035	0.842	0.898	0.771	0.047
LSR_21_^11^	0.787	0.838	0.696	0.080	0.804	0.880	0.673	0.037	0.840	0.895	0.766	0.048
R-MGL_21_^13^	0.775	0.812	0.673	0.088	0.814	0.852	0.666	0.035	0.833	0.867	0.740	0.052
PFNet_21_^29^	0.782	0.841	0.695	0.085	0.800	0.877	0.660	0.040	0.829	0.887	0.745	0.053
C2FNet-V2_22_^55^	0.799	0.859	0.730	0.077	0.811	0.887	0.691	0.036	0.840	0.896	0.770	0.048
ERRNet_22_^56^	0.779	0.842	0.679	0.085	0.786	0.867	0.630	0.043	0.827	0.887	0.737	0.054
TPRNet_22_^32^	0.807	0.861	0.725	0.074	0.817	0.887	0.683	0.036	0.846	0.898	0.768	0.048
FAPNet_22_^26^	0.815	0.865	0.734	0.076	0.822	0.888	0.694	0.036	0.851	0.899	0.775	0.047
BGNet_22_^58^	0.812	0.870	0.749	0.073	0.831	0.901	0.722	0.033	0.851	0.907	0.788	0.044
PreyNet_22_^28^	0.790	0.842	0.708	0.077	0.813	0.881	0.697	0.034	0.834	0.887	0.763	0.050
ZoomNet_22_^27^	0.820	0.877	0.752	0.066	0.838	0.888	0.729	0.029	0.853	0.896	0.784	0.043
SINetV2_22_^50^	0.820	0.882	0.743	0.070	0.815	0.887	0.680	0.037	0.847	0.903	0.770	0.048
DGNet_23_^19^	0.839	0.901	0.769	0.057	0.822	0.896	0.693	0.033	0.857	0.911	0.784	0.042
FSPNet_23_^59^	0.856	0.899	0.799	0.050	0.851	0.895	0.735	0.026	0.879	0.915	0.816	0.035
Camoformer-C_24_^60^	0.859	0.913	0.812	0.050	0.860	0.926	0.770	0.024	0.883	0.933	0.788	0.032
MSCNet_24_^61^	0.873	0.927	0.826	0.046	0.861	0.925	0.770	0.025	0.884	0.931	0.833	0.033
CINet_24_^62^	0.847	0.899	0.794	0.055	0.841	0.914	0.744	0.028	0.868	0.924	0.815	0.037
SDRNet_24_^63^	0.872	0.924	0.826	0.049	0.871	0.924	0.785	0.023	0.889	0.934	0.842	0.032
MIGNet_25_^64^	0.875	0.926	0.831	0.044	0.861	0.926	0.768	0.025	0.885	0.930	0.836	0.033
Ours	0.882	0.931	0.842	0.044	0.869	0.930	0.785	0.023	0.890	0.935	0.844	0.032

#### Qualitative analysis

**Fig. 6:**
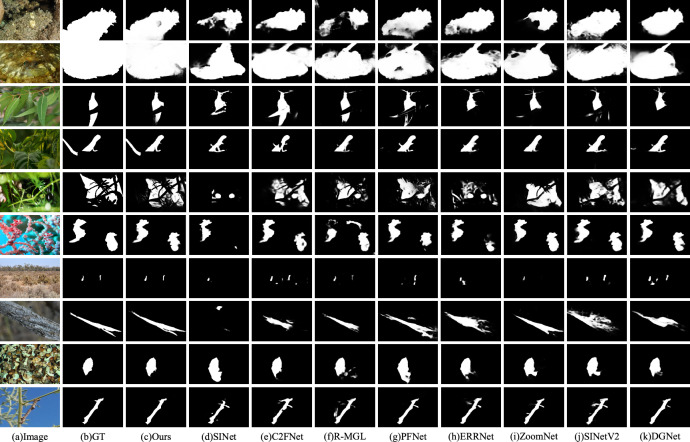
Qualitative comparison of the proposed method with other methods (i.e., SINet, C2FNet, R-MGL, PFNet, ERRNet, ZoomNet, SINetV2, DGNet). (The images are sourced from publicly available COD datasets: COD10K (https://github.com/DengPingFan/SINet/), NC4K (https://github.com/JingZhang617/COD-Rank-Localize-and-Segment),and CAMO (https://sites.google.com/view/ltnghia/research/camo). All datasets are available for non-commercial use, and only require citation of the corresponding papers).

Figure 6 illustrates the visual comparison results for the 10 test samples in the baseline dataset for comparison. These samples encompass various challenges such as big objects, occlusion, small objects, background matching, and mimicry problems. Here, we present the detection results for SINet, C2FNet, R-MGL, PFNet, ERRNet, ZoomNet, SINetV2, DGNet, and our method. The compared methods tend to exhibit inaccurate object localization, incomplete object areas, or even missing objects, leading to poor segmentation of camouflaged objects. As shown in rows 1 and 2 of the figure, our method accurately delineates big objects, providing more complete object regions compared to other methods. Additionally, for camouflaged objects with varying degrees of occlusion (rows 3, 4, and 5), our model demonstrates the ability to identify them accurately and determine a complete individual of objects without failing to complete clustering due to occlusion. In row 3, both C2FNet and R-MGL are capable of distinguishing the upper and lower parts as a single object, but there are false detections as well as missed detections. In scenarios involving multiple objects or small objects (rows 6 and 7), our model demonstrates effective localization and segmentation of the objects. Furthermore, even in cases of background matching or mimicry, our model maintains the capability to recognize camouflaged objects effectively. These results demonstrate the robustness of the method across various challenging scenarios.

### Ablation study

Table 2 presents the ablation results of the network. We conducted comprehensive ablation experiments on the MFAM and HMIM to validate the contribution of each key component of our approach. Specifically, our ablation study involves six main models. In the Basic (M1) experiment, we removed all the proposed modules and retained only the backbone model. In the Basic + MFAM (M2) experiment, we added the MFAM to M1. In the Basic + w/o HMIM (M3) experiment, we added the HMIM module to M1 while removing the texture fusion part, retaining only the feature modulation part. In the Basic + MFAM + w/o HMIM (M4) experiment, we added the content of the MFAM to M3. In Basic + HMIM (M5), we implemented the complete content of the HMIM module, including the texture feature fusion part. In Basic + HMIM + HMIM (M6), we present our complete model.

**Table 2:** Results of the quantitative evaluation of different ablation models on three benchmark datasets using the four metrics $S_{\alpha }$, $E_{\phi }$, $F_{\beta }^{\omega }$ and $M$. “${ \uparrow \mathord{\left/ {\vphantom { \uparrow \downarrow }} \right. \kern-0pt} \downarrow }$” indicates that bigger or smaller is better.

Baseline models	CAMO				COD10K				NC4K			
	$S_{\alpha } \uparrow$	$E_{\phi } \uparrow$	$F_{\beta }^{\omega } \uparrow$	$M \downarrow$	$S_{\alpha } \uparrow$	$E_{\phi } \uparrow$	$F_{\beta }^{\omega } \uparrow$	$M \downarrow$	$S_{\alpha } \uparrow$	$E_{\phi } \uparrow$	$F_{\beta }^{\omega } \uparrow$	$M \downarrow$
Basic (M1)	0.854	0.902	0.771	0.059	0.836	0.898	0.692	0.034	0.870	0.915	0.783	0.042
Basic + MFAM (M2)	0.863	0.914	0.806	0.053	0.855	0.920	0.751	0.027	0.881	0.929	0.821	0.035
Basic + w/o HMIM (M3)	0.869	0.912	0.809	0.051	0.860	0.921	0.758	0.026	0.886	0.928	0.829	0.034
Basic + MFAM + w/o HMIM (M4)	0.878	0.930	0.835	0.045	0.867	0.928	0.780	0.024	0.889	**0.936**	0.842	0.032
Basic + HMIM (M5)	0.877	0.928	0.833	0.046	0.867	0.927	0.778	0.024	0.889	0.935	0.841	0.032
Basic + HMIM + MFAM (M6)	**0.882**	**0.931**	**0.842**	**0.044**	**0.869**	**0.930**	**0.785**	**0.023**	**0.890**	0.935	**0.844**	**0.032**

The best results are highlighted in bold.

#### Effectiveness of MFAM

**Fig. 7:**
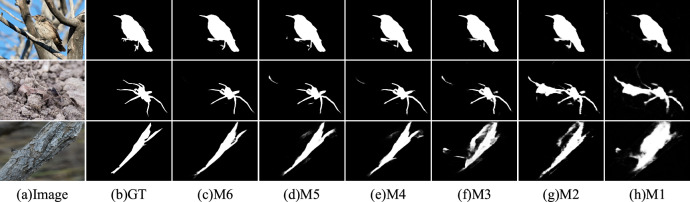
Visual comparison of detection results obtained with different models in ablation studies. (The images are sourced from publicly available COD datasets: COD10K (https://github.com/DengPingFan/SINet/), NC4K (https://github.com/JingZhang617/COD-Rank-Localize-and-Segment),and CAMO (https://sites.google.com/view/ltnghia/research/camo). All datasets are available for non-commercial use, and only require citation of the corresponding papers).

The effectiveness of MFAM can be verified by comparing three pairs of models: M1 to M2, M3 to M4, and M5 to M6. In all three cases, the inclusion of the MFAM significantly enhances detection accuracy in the model. From Fig. 7, it is evident that the model containing MFAM can more effectively filter out the noise interference than the model without MFAM.

#### Effectiveness of HMIM

From experiments M1 to M3 and M2 to M4, it can be observed that even though the HMIM module only contains the feature modulation part, it still exhibits varying degrees of improvement in performance. This indicates the effectiveness of our grouping strategy and progressive fusion approach. After this module complements the fusion part of the texture features, the results from M3 to M5 and M4 to M6 demonstrate that the model’s performance improves to varying degrees. This validates that the utilization of texture information contributes to the performance of the camouflage object detection model. This can also be observed in Fig. 7.

#### Number of groups in HMIM

In Table 3, we demonstrate the effect of different branch numbers on the proposed HMIM to validate the rationality of the HMIM hierarchical structure. By comparing 1, 2, and 4 branches, we observe that the model performs better on these datasets with increased branches. This improvement is attributed to the enhanced fusion of contextual information with texture information as the number of branches increases, leading to better performance. However, this trend disappears in the 6-branch variant, as the model tends to overfit with the increase in parameters due to the finite nature of the training data. In summary, the best performance is achieved when the number of groups is 4. Moreover, it strikes a good balance between performance and efficiency. To be fair, we set the number of groups in each HMIM to 4 in other experiments.

**Table 3:** An ablation study on the structural rationality of HMIM. We present four hierarchies: 1, 2, 4, and 8.

Branch	CAMO				COD10K				NC4K			
	$S_{\alpha } \uparrow$	$E_{\phi } \uparrow$	$F_{\beta }^{\omega } \uparrow$	$M \downarrow$	$S_{\alpha } \uparrow$	$E_{\phi } \uparrow$	$F_{\beta }^{\omega } \uparrow$	$M \downarrow$	$S_{\alpha } \uparrow$	$E_{\phi } \uparrow$	$F_{\beta }^{\omega } \uparrow$	$M \downarrow$
1	0.874	0.926	0.827	0.047	0.862	0.925	0.768	0.025	0.887	0.934	0.836	0.033
2	0.879	0.928	0.833	0.046	0.868	0.927	0.780	0.024	0.890	0.934	0.841	0.032
4	**0.882**	**0.931**	**0.842**	**0.044**	**0.869**	**0.930**	**0.785**	**0.023**	**0.890**	0.935	**0.844**	**0.032**
6	0.878	0.931	0.838	0.045	0.867	0.930	0.784	0.023	0.889	**0.936**	0.844	0.032

The best results are highlighted in bold.

#### Effectiveness of MFAM & HMIM

Table 2 demonstrates that the complete structure of the proposed model generally performs better than the other setups. This illustrates the mutually reinforcing role of the two components. Compared to the M1 baseline, On the COD10K dataset, it achieved improvements of 3.9% and 13.4% for $S_{\alpha }$ and $F_{\beta }^{\omega }$, respectively, and reduced $M$ by 32.4%. These explicit performance gains originate entirely from our custom-designed modules (HMIM and MFAM) rather than the PVTv2. The best results are also evident in Fig. 7. These results indicate that the complete structure and the cascade approach presented in this paper are more favorable for detecting camouflaged objects.

#### Computational efficiency

To evaluate the computational complexity of the model, we conducted a comparative analysis with various model variants generated through ablation experiments in Table 4. This study employs the number of floating-point operations (FLOPs) and parameters (Params) as metrics for measuring model complexity. Experimental results show that compared to the baseline model, the full model’s parameter count increased from 25 to 28 M—a relatively modest growth. Meanwhile, FLOPs rose from 24.2G to 42.3G, reflecting a higher computational load. Notably, as each functional module was progressively introduced, the increase in model complexity was accompanied by consistent performance improvements. For example, the introduction of the HMIM module led to a significant rise in FLOPs, but it also contributed to a substantial gain in model performance. This demonstrates that the computational overhead introduced by this module is both effective and necessary. In terms of inference efficiency, the complete model maintains a processing speed exceeding 30 FPS, meeting real-time requirements. However, we acknowledge that there remains room for further improvement in computational efficiency.

**Table 4:** Compare FLOPs and Params among dissolution models.

Method	M1	M2	M3	M4	M5	M6
Params(M)	25.440	25.675	27.929	28.164	28.037	28.272
FLOPs(G)	24.253	25.695	39.125	40.567	40.858	42.300
FPS	72.933	46.735	53.330	37.923	42.572	31.206

### Application to Polyp segmentation

Camouflaged object detection has rich downstream applications. Here, we show the performance of our network in terms of polyp segmentation. To identify polyps from colonoscopy pictures for real-time resection, polyp segmentation is crucial. In this experiment, we use five publicly available datasets, including Kvasir^66^, CVC-ClinicCB^67^, ETIS^68^, CVC-ColonDB^43^, and CVC-T^69^. We followed the same experimental setup^1,70^, that 900 and 550 images were collected from Kvasir and ClinicCB datasets, respectively, to compose the training set. The remaining images, along with those from three additional datasets, constitute the test set. We compare the performance of our model with five representative state-of-the-art polyp segmentation models: UNet^45^, UNet +  + ^46^, SFA^71^, PraNet^1^, and MSNet^70^. We employed six widely used metrics in polyp segmentation, including mean Dice and mean IoU, as well as $S_{\alpha }$, $F_{\beta }^{\omega }$, $E_{\phi }^{\max }$, and $M$ to accomplish quantitative evaluation.

#### Quantitative analysis

In Table 5, we compare the five state-of-the-art methods using six evaluation metrics across these five datasets. Our method outperforms the other methods on all datasets, with clear advantages. Fully optimal results are achieved on the Kvasir, ColonDB, and CVC-T datasets. For instance, on the ColonDB dataset, our network demonstrates improvements of 4%, 7.8%, 6.4%, 4.7%, 3.2%, and 29.3% on mDice, mIoU, as well as $S_{\alpha }$, $F_{\beta }^{\omega }$, $E_{\phi }^{\max }$, and $M$, respectively, compared to the second-best method, MSNet. In addition, we still achieve superior results on the ClinicDB and ETIS datasets. On the ETIS dataset, our network exhibits improvements of 7.9%, 5.7%, 7.7%, 3.7%, and 10% on mDice, mIoU, as well as $S_{\alpha }$, $F_{\beta }^{\omega }$, and $E_{\phi }^{\max }$, respectively, compared to the second-best method, MSNet.

**Table 5:** Quantitative evaluation of polyp segmentation on five datasets.

Method		${\mathrm{mDice}} \uparrow$	${\mathrm{mIoU}} \uparrow$	$F_{\beta }^{\omega } \uparrow$	$S_{\alpha } \uparrow$	$E_{\phi }^{\max } \uparrow$	${\mathrm{MAE}} \downarrow$
Kvasir	UNet	0.818	0.746	0.794	0.858	0.893	0.055
	UNet + +	0.821	0.743	0.808	0.862	0.910	0.048
	SFA	0.723	0.611	0.670	0.782	0.849	0.075
	ParNet	0.898	0.840	0.885	0.915	0.948	0.030
	MSNet	0.907	0.862	0.893	0.922	0.944	0.028
	Ours	**0.917**	**0.867**	**0.908**	**0.929**	**0.965**	**0.025**
ClinicDB	UNet]	0.823	0.755	0.811	0.889	0.954	0.019
	UNet + +	0.794	0.729	0.785	0.873	0.931	0.022
	SFA	0.700	0.607	0.647	0.793	0.885	0.042
	ParNet	0.899	0.849	0.896	0.936	0.979	0.009
	MSNet	0.921	**0.879**	0.914	0.941	0.972	0.008
	Ours	**0.921**	0.871	**0.915**	**0.946**	**0.979**	**0.007**
ETIS	UNet	0.398	0.335	0.366	0.684	0.740	0.036
	UNet + +	0.401	0.344	0.390	0.683	0.776	0.035
	SFA	0.297	0.217	0.231	0.577	0.633	0.109
	ParNet	0.628	0.567	0.600	0.794	0.841	0.031
	MSNet	0.719	0.664	0.678	0.840	0.830	**0.020**
	Ours	**0.776**	**0.702**	**0.730**	**0.871**	**0.913**	0.021
ColonDB	UNet	0.512	0.444	0.498	0.712	0.776	0.061
	UNet + +	0.483	0.410	0.467	0.691	0.760	0.064
	SFA	0.469	0.347	0.379	0.634	0.765	0.094
	ParNet	0.709	0.640	0.696	0.819	0.869	0.045
	MSNet	0.755	0.678	0.737	0.836	0.883	0.041
	Ours	**0.806**	**0.731**	**0.784**	**0.875**	**0.911**	**0.029**
CVC-T	UNet	0.710	0.627	0.684	0.843	0.876	0.022
	UNet + +	0.707	0.624	0.687	0.839	0.898	0.018
	SFA	0.467	0.329	0.341	0.640	0.817	0.065
	ParNet	0.871	0.797	0.843	0.925	0.972	0.010
	MSNet	0.869	0.807	0.849	0.925	0.943	0.010
	Ours	**0.894**	**0.829**	**0.880**	**0.939**	**0.976**	**0.006**

The best results are highlighted in bold.

#### Qualitative analysis

**Fig. 8:**
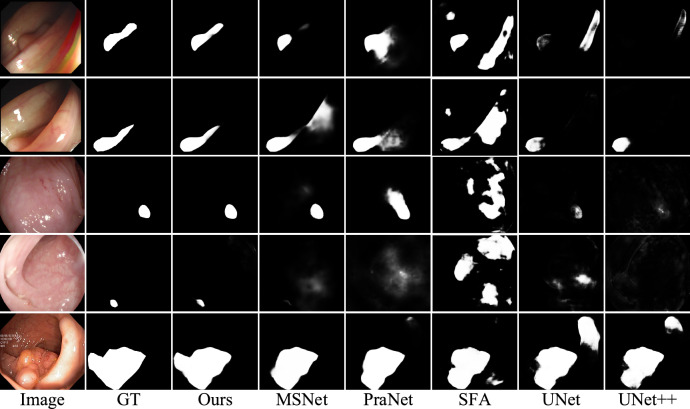
Visual comparison among different methods in a polyp segmentation task. (The images are sourced from publicly available poly segmentation datasets: Kvasir(https://github.com/DebeshJha/Kvasir-SEG), CVC-ClinicDB(https://polyp.grand-challenge.org/CVCClinicDB/), ETIS(https://service.tib.eu/ldmservice/dataset/etis-larib-polyp-db), CVC-ColonDB(http://vi.cvc.uab.es/colon-qa/cvccolondb/), and CVC-T(https://gitcode.com/open-source-toolkit/9a017).All datasets are available for non-commercial use, and only require citation of the papers).

Figure 8 illustrates the segmentation results of our model compared to the other six models. As can be seen from the visual results, our model produces segmentation results that are closer to the ground truth map. In the first, second, and fourth rows, polyps exhibit different shapes and larger sizes, posing challenges for accurate segmentation. Our model successfully segments their contours completely. In the third and fourth rows, due to the visual embedding of polyps in their surroundings, an unclear boundary between the polyp and the background poses a significant challenge for segmentation and identification. In some cases, PraNet and SFA failed to segment the complete location of the polyp or even exhibited incorrect segmentation results. Overall, the visual results provide additional evidence of our method’s ability to handle polyp segmentation tasks.

## Conclusion

In this paper, we propose a novel camouflage object detection framework CTHINet. To achieve effective camouflage object detection, our proposed framework adopts a two-branch structure. This structure is designed to extract multi-scale context-aware and texture information separately, mitigating interference between the underlying information. Furthermore, we introduce an MFAM to hierarchically extract features with different receptive field sizes within a single feature utilizing multiple heads. This module is designed to leverage layering operations, providing models with the capability to match candidate areas of camouflaged objects with varying sizes and shapes. In addition, we have designed an HMIM, enabling the effective coupling of multi-scale information with texture information. Experimental results on three datasets demonstrate that our proposed CTHINet outperforms existing COD methods. Additionally, a comprehensive evaluation of the polyp segmentation dataset reveals the promising potential of CTHINet for downstream applications.

## Data Availability

The data that support the findings of this study are available from the corresponding author, Xiaoxia Lu (huaihe_radiology@163.com), upon reasonable request.
